# Refractory Ventricular Fibrillation in Traumatic Cardiac Arrest: A Case Report and Review of the Literature

**DOI:** 10.7759/cureus.19851

**Published:** 2021-11-24

**Authors:** Mohammed Alageel, Nawaf A Aldarwish, Faisal A Alabbad, Fahad M Alotaibi, Mohammed N Almania, Saad M Alshalawi

**Affiliations:** 1 Emergency Medicine and Critical Care, King Saud University, Riyadh, SAU; 2 Emergency Medicine, University of British Columbia, Faculty of Medicine, Vancouver, CAN; 3 Medicine, King Saud University, Riyadh, SAU

**Keywords:** traumatic cardiac arrest., emergency thoracotomy, ventricular fibrillation, refractory, trauma

## Abstract

Ventricular fibrillation (VF) is a lethal cardiac arrhythmia that leads to cardiac arrest and death. It is especially deadly when it fails to respond to conventional treatment with electrical defibrillation. This arrhythmia is often triggered by acute myocardial ischemia, but in rare cases, it can be precipitated by direct myocardial trauma. Most patients with traumatic cardiac arrest do not survive, but in a minority of patients, an emergency thoracotomy may improve survival by addressing reversible causes such as haemorrhage control, relief of cardiac tamponade, and direct wound closure. We present an unusual case of a traumatic cardiac arrest, presenting with refractory ventricular fibrillation due to a cardiac laceration in a young trauma patient with an isolated chest injury.

## Introduction

Traumatic cardiac arrest is defined as a cardiac arrest resulting from an external application of kinetic energy [1]. Ventricular fibrillation (VF) is a life-threatening cardiac arrhythmia that causes loss of heart function and sudden death. On an electrocardiogram (ECG), VF is defined as a disorganized and irregular electrical activity with no discernable pattern [2]. Inspection of the ventricles would show them to be quivering rather than beating [3]. Refractory ventricular fibrillation (RVF) is described as the inability to obtain a return of spontaneous circulation (ROSC) within 10 min despite three defibrillation attempts, 300 mg of amiodarone, and 3 mg of epinephrine [4]. Trauma can present in multiple forms, and any of them can lead to a potentially reversible cause of cardiac arrest, from hypoxia, tension pneumothorax, hypovolaemia, or cardiac tamponade [5-6]. The pathophysiological mechanisms of VF are due to electrical reentry or increased automaticity, which is often a result of myocardial injury from an acute coronary syndrome (ACS). Other causes include electrolyte abnormalities such as abnormalities in serum potassium levels, drugs that cause myocardial toxicity, and underlying genetic diseases that affect the heart's ionic channels or electrical conduction [7]. Ventricular fibrillation has been reported in multiple case reports due to traumatic injuries [8-9].

The mainstay of treatment of cardiac arrest due to VF is highlighted in the Advanced Cardiovascular Life Support (ACLS) algorithm, which consists of high-quality cardiopulmonary resuscitation (CPR), accurate heart rhythm diagnosis, and early defibrillation for cases of VF and pulseless ventricular tachycardia, and intravenous administration of epinephrine and antiarrhythmic drugs [10].

## Case presentation

**Figure 1:**
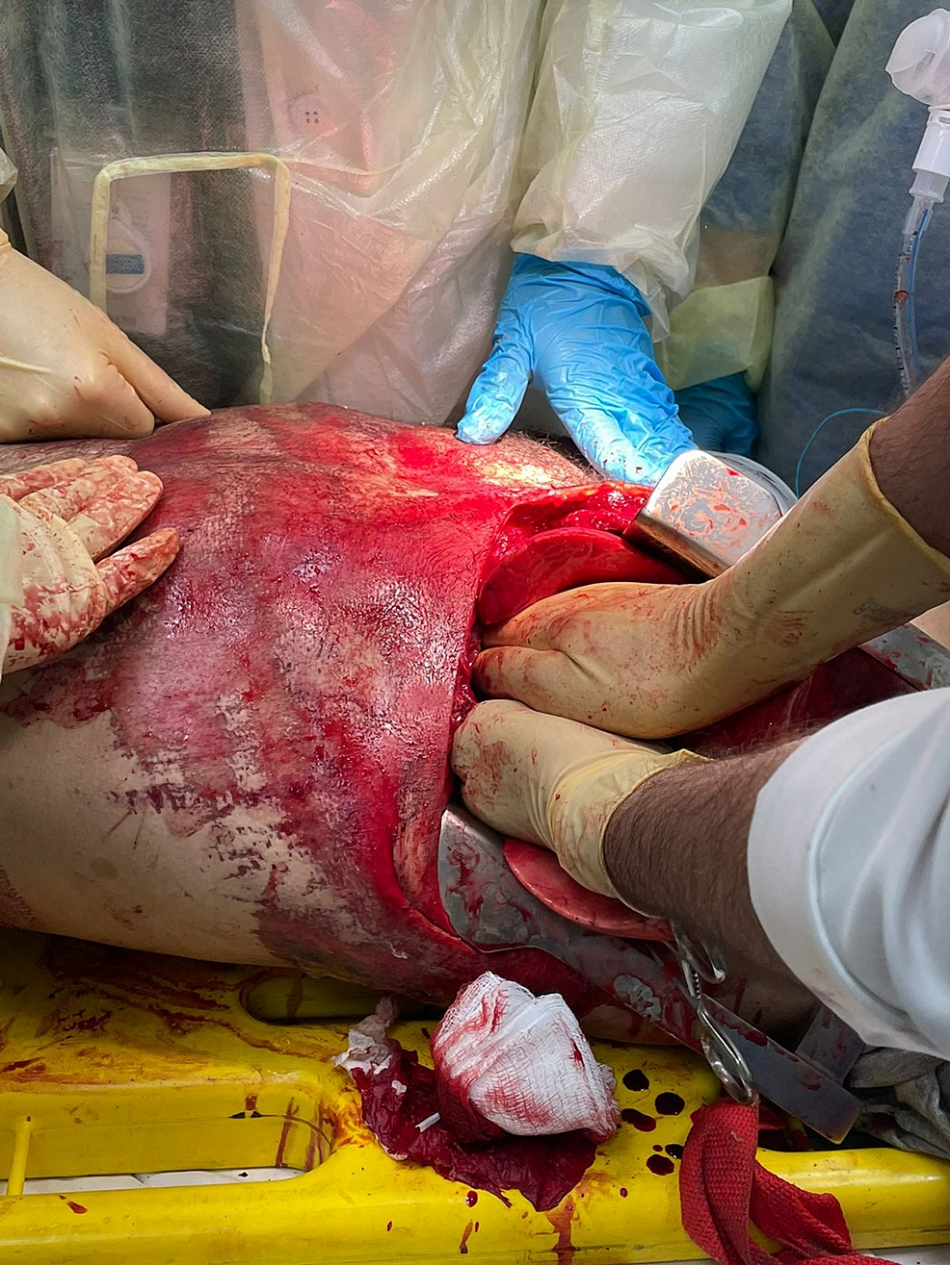
Left lateral emergency thoracotomy with delivery of the heart and open cardiac massage

**Figure 2:**
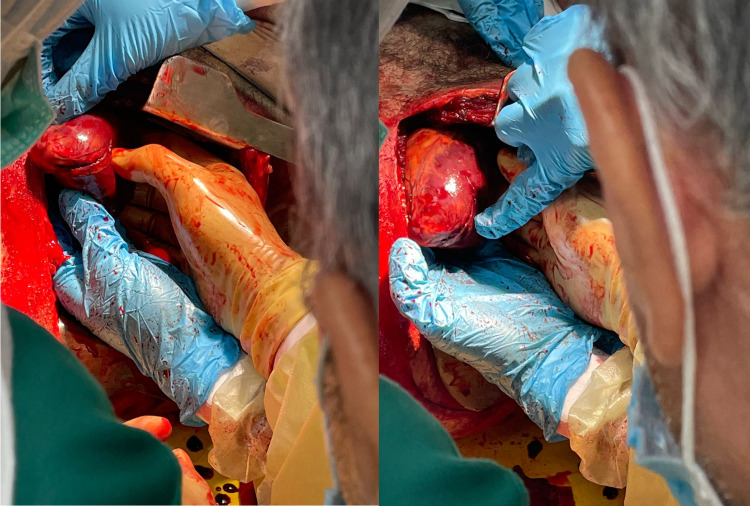
Delivered heart demonstrating longitudinal laceration across the ventricles

A 29-year-old male was brought in by emergency medical services (EMS) after being involved in a motor vehicle collision. He was found on the floor next to a collided vehicle with no other passengers. He was unresponsive, with weak respiratory efforts but a palpable pulse. The patient was transported to a nearby emergency department (ED) and upon arrival in the ambulance bay, the patient's respiratory efforts ceased and cardiopulmonary resuscitation (CPR) was initiated by the EMS crew. In the ED, with obvious signs of a traumatic chest injury and a deformed chest wall with multiple superficial lacerations, he underwent placement of bilateral chest tube thoracostomies after bilateral finger decompression of the chest cavities. His bedside echocardiogram demonstrated a cardiac standstill with no pericardial effusion corresponding to an initial rhythm of asystole on the electrocardiographic monitor. The patient was continued on the standard ACLS protocol while the airway was secured and intravenous access was established. At 10 minutes after presentation, an emergency thoracotomy was performed in the left lateral aspect of the chest (Figure 1). The thoracotomy did not demonstrate significant intrathoracic bleeding within the chest cavity, nor any obvious lung injuries. There were, however, multiple rib fractures with fragments within the thoracic cavity. The heart was delivered from the pericardium with no surrounding effusion, a longitudinal nonpenetrating heart laceration was observed over the left ventricle (Figure 2). The ventricles appeared to be fluctuating on palpation and the cardiac monitor demonstrated fine ventricular fibrillation (VFIB). Manual ​​cardiac massage was initiated, and the patient received conventional chest wall unsynchronized cardioversion due to lack of intracardiac pedals and open chest wall, and ACLS was continued with the addition of two doses of intracardiac epinephrine, and a clamp was applied to the descending thoracic aorta. During the resuscitation period, the patient had received a normal saline bolus through intraosseous access, as well as one liter of un-crossmatched blood through a central femoral line.

Notably, the abdominal-focused assessment of sonography for trauma (FAST) was performed twice, which were negative in both. The patient had no obvious signs of head injury, both pupils were fixed and dilated, the pelvis was stable on examination, no external bleeding or long bone injuries were observed, and the cardiac ventricles demonstrated good filling on manual cardiac massage. After 25 minutes of ACLS with cardiac massage, including multiple defibrillation attempts, and administration of the antiarrhythmic drugs, the patient remained in refractory ventricular fibrillation. Resuscitation efforts were halted, and the patient was pronounced dead.

## Discussion

Cardiac arrest due to medical diseases, such as myocardial infarction, is the most common etiology. Traumatic cardiac arrest (TCA) is an uncommon cause of cardiac arrest and is associated with worse outcomes [11]. The reported survival rate of a traumatic out-of-hospital cardiac arrest is 1% to 7% [6]. However, there is increasing evidence that when potentially reversible causes are addressed early in the adult population, TCA survival rates are comparable to those reported in medical out-of-hospital cardiac arrests [12].

The incidence of refractory VF is estimated to be between 0.5 and 0.6 per 100,000 persons [2]. When refractory VF presents, mortality rates are estimated to be between 85% and 97% [8].

Thoracotomy's role in patients who had a blunt traumatic injury is less well-defined, but there is growing evidence that an emergency thoracotomy can provide access to the heart to relieve traumatic pericardial effusions, facilitate closure of simple wounds (e.g. left auricular appendage lacerations), enable vascular control of the thoracic aorta, which can be achieved by simple compression to reduce bleeding below the diaphragm, provide access to the hemithoraces to enable bleeding control measures of the lung (e.g. by collapse or twisting of a lung), and permit internal cardiac compression to be performed [13-14].

In this case, we followed the standard approach for treating a traumatic cardiac arrest, although there are more novel proposed interventions with growing evidence that may have been attempted such as extracorporeal membrane oxygenation (ECMO) and an esmolol infusion. In cardiac arrest, the use of ECMO has been associated with an increased survival rate, as well as an increase in favorable neurological outcomes [9,15]. Unfortunately, the most common complication from ECMO is bleeding due to the anticoagulation requirements to maintain a functional circuit [16]. In patients with traumatic injuries, hemorrhage is the most common cause of mortality [17]. Thus, the decision to use ECMO in patients with traumatic injuries can pose a dilemma in clinical practice. However, one cohort study indicated that critical care patients with traumatic injuries receiving ECMO support might have more favorable survival outcomes compared to other critically ill patients [18]. Data on the optimal strategy for the use of anticoagulation in ECMO is limited and mostly expert opinion based, with unfractionated heparin being recommended [19]. Another proposed therapy for refractory VFIB is Esmolol, a strong beta-1 antagonist, which has been found to improve return of spontaneous circulation (ROSC), increase the number of successful defibrillation attempts, and improve the duration of post-resuscitation survival in refractory VF. The theoretical mechanism of action in refractory VF is thought to be due to blocking the high exogenous and endogenous catecholamine state of cardiac arrest [20].

## Conclusions

In traumatic cardiac arrest, early and invasive interventions may improve low survival rates by treating reversible causes in addition to the conventional ACLS algorithms. The conventional management of this case, which consisted of CPR, early defibrillation, and emergency thoracotomy, was carried out, however, the patient did not survive. This raises the question of offering other, more novel measures such as ECMO and an Esmolol infusion.

Although several incidences of traumatic cardiac arrest that resulted in ventricular fibrillation are documented, to the best of our knowledge, this is the first case of refractory ventricular fibrillation resulting from a traumatic cardiac injury.
